# Diverse platforms, diverse effects: Evidence from a 100-day study on social media and adolescent mental health

**DOI:** 10.1007/s12144-025-08893-7

**Published:** 2025-12-19

**Authors:** Amber van der Wal, Ine Beyens, Loes H. C. Janssen, Patti M. Valkenburg

**Affiliations:** 1 Amsterdam School of Communication Research, University of Amsterdam, Amsterdam, P.O. Box 15791, NG 1001 The Netherlands; 2 Present Address: Department of Developmental Psychology, University of Utrecht, Heidelberglaan 1, 3584 CS Utrecht, The Netherlands

**Keywords:** Social media, Adolescents, Mental health, Well-Being, Person-Specific effects

## Abstract

The rising prevalence of mental health problems among adolescents has prompted increased scrutiny of social media as a contributing factor. Previous research has produced mixed results, likely due to the varying impact of social media on different dimensions of mental health. To advance understanding in this area, this study examined how social media use affects three critical dimensions of adolescent mental health – well-being, self-esteem, and friendship closeness. Specifically, we examined whether adolescents experienced consistent (unity) or contrasting (duality) effects across these dimensions by analyzing 44,211 daily diaries from 479 adolescents over 100 days. We found that the majority of adolescents (60%) experienced unity in negative effects of social media, suggesting that social media use is a notable contributor to mental health issues. Moreover, 13.6% of adolescents experienced duality in effects, indicating that social media use simultaneously harms and benefits different dimensions of their mental health. Exploratory analyses demonstrated the importance of examining platform-specific effects, revealing negative impacts of TikTok, YouTube, and Instagram use and positive or null effects of Snapchat and WhatsApp use on the three dimensions of mental health. Our findings highlight the need for tailored strategies that account for the varying impacts of social media on adolescent mental health.

The global adolescent population today totals 1.3 billion (World Health Organization, 2023). An estimated 14% of these adolescents, which translates to approximately 182 million individuals, are grappling with mental health problems (Institute of Health Metrics and Evaluation, 2020). Moreover, the prevalence of mental health problems among adolescents continues to rise in many countries (Keyes & Platt, 2024). In response to what is widely regarded as a global mental health crisis, the World Health Organization (WHO) has prioritized the promotion of adolescent mental health. Three essential dimensions of adolescents’ mental health that need to be promoted are affective well-being, which is characterized by experiencing more positive than negative emotions (Kross et al., 2021, referred to as well-being from hereon), self-esteem, reflected in establishing and maintaining a positive self-view (Rosenberg, 1965), and having close friendships (Raboteg-Saric & Sakic, 2014).

A key factor believed to impact adolescents’ well-being, self-esteem, and closeness of their friendships, is their social media use. Yet, social media’s effects on youth mental health remain highly contested, with some arguing that social media are the primary driver of the youth mental health crisis (Haidt, 2020; Twenge et al., 2022), while others contend that their impact is negligible (Ferguson et al., 2025) – or even beneficial (Godard & Holtzman, 2024). Qualitative studies with adolescents suggest that the effects depend on the specific mental health dimension being considered (Keles et al., 2024; Shankleman et al., 2021). Quantitative research further supports this dimension-specific perspective. For example, review studies and meta-analyses indicate a weakly negative relationship between social media use and self-esteem (Saiphoo et al., 2020), a near-zero relationship with well-being (Hancock et al., 2022; Huang, 2017), and a moderately positive one with friendship closeness (Liu et al., 2016).

In addition to being dimension-specific, a growing body of research underscores the person-specific nature of social media’s impact on mental health. These relatively new studies track changes within individuals over time, revealing substantial heterogeneity in the effects of social media use on various dimensions of mental health, indicating that its impact is highly variable across individuals (Griffioen et al., 2023; Karsay et al., 2023; Marciano et al., 2022). Importantly, pronounced person-specific effects of social media have also been found for the three dimensions of mental health that the current study focuses on – well-being (Beyens et al., 2024), self-esteem (Valkenburg et al., 2021), and friendship closeness (Pouwels et al., 2021). For instance, Beyens et al. (2024) demonstrated that the effect of social media on well-being is negative for 28% of adolescents, positive for 26%, and negligible for nearly half.

Guided by previous studies that have highlighted variability in how social media use impacts different individuals and dimensions of mental health, we argue that the impact of social media use on mental health may be both dimension-specific and person-specific. However, no research has systematically examined how effects may vary within single individuals across multiple dimensions. For instance, it is unknown whether adolescents who experience negative effects of social media on well-being (Beyens et al., 2024) also experience negative effects on self-esteem and friendship closeness. Therefore, the overarching goal of this study is to understand how social media’s impact on well-being, self-esteem, and friendship closeness may differ not only from adolescent to adolescent but also within each individual adolescent. By being the first to investigate these person-specific and dimension-specific effects simultaneously, this study will provide critical insights for developing tailored recommendations, policies, and interventions.

To achieve a robust examination of dimension- and person-specific effects of social media on adolescent mental health, we implemented an intensive longitudinal design spanning 100 days, where each evening, participants completed a micro-questionnaire via their smartphones, reporting on their daily social media use as well as their well-being, self-esteem, and friendship closeness^1^. This approach offers three key advancements over previous studies. First, while much research has examined long-term effects using yearly intervals (e.g., Coyne et al., 2020; Tandoc & Goh, 2021; Valkenburg et al., 2017), such extended timeframes do not capture the short-term fluctuations through which social media likely exerts its effects. Second, existing short-term studies tracked participants for one (Karsay et al., 2023), two (Colasante et al., 2024; Irmer & Schmiedek, 2023) or three weeks (Beyens et al., 2024; Pouwels et al., 2021; Valkenburg et al., 2021). Third, many earlier studies (Beyens et al., 2024; Karsay et al., 2023; Pouwels et al., 2021; Valkenburg et al., 2021) were conducted prior to the shift towards AI-driven social media platforms (such as TikTok), which use algorithms to provide a continuous stream of personalized content, substantially altering the social media landscape.

## Within-person unity and duality

To facilitate an understanding of the potentially differential impacts of social media use on adolescents’ well-being, self-esteem, and friendship closeness, we introduce a novel framework centered on the concepts of “within-person unity” and “within-person duality.” This framework provides a new theoretical perspective on whether social media effects are homogeneous or heterogeneous across multiple mental health dimensions within individual adolescents. Within-person unity reflects homogeneous effects, where adolescents experience uniformly positive, negative, or no effects of social media use across dimensions of mental health. In contrast, within-person duality reflects heterogeneous, diverging effects, where social media use has contrasting impacts on different mental health dimensions. For example, an adolescent may experience positive effects of social media use on friendship closeness while simultaneously experiencing negative effects on well-being and self-esteem.

The within-person unity/duality framework is conceptually grounded within well-established theories that emphasize the multifaceted nature of media use and its potential implications for key dimensions of adolescent mental health. For instance, uses and gratifications theory (Katz et al., 1973) and self-determination theory (Deci & Ryan, 2012) both suggest that individuals engage with media to fulfill core psychological and social needs. Building on this notion, within-person unity may emerge when social media use uniformly supports or undermines these needs across domains, while within-person duality may occur when certain needs are fulfilled (e.g., the need for entertainment), but others are simultaneously frustrated (e.g., the need for social interaction). These consistent or conflicting patterns of need fulfillment may, in turn, manifest as uniform or divergent effects on adolescents’ well-being, self-esteem, and friendship closeness.

Existing research offers initial, yet indirect, support for the relevance of the within-person unity/duality framework. For instance, earlier research has found strong bivariate correlations between well-being, self-esteem, and friendship closeness (Verbeij et al., 2022), suggesting that a substantial subset of adolescents may experience unity in the effects of social media use on these mental health dimensions. At the same time, qualitative research suggests that adolescents often report both positive and negative effects of social media use on their mental health (van der Wal et al., 2024; Weinstein, 2018), consistent with the notion of within-person duality. However, these studies fall short of capturing how often such duality occurs, or how it unfolds across multiple dimensions of mental health simultaneously. By formally conceptualizing within-person unity and duality, the present study offers a theoretical and empirical foundation for more precise and differentiated insights into how adolescents experience social media in their everyday lives.

To apply the within-person unity/duality framework, we examine the prevalence of three types of within-person unity and three types of within-person duality (RQ1). Within-person unity occurs when adolescents experience exclusively or predominantly uniform (non-contrasting) effects across dimensions, which can manifest in three ways: (1) null effects across all three dimensions, (2) negative effects across all three dimensions or negative effects in two dimensions with a null effect in the remaining dimension, or (3) positive effects across all three dimensions or positive effects in two dimensions with a null effect in the remaining dimension. Conversely, within-person duality involves contrasting effects and can manifest in three ways: (1) two positive effects combined with one negative effect, (2) two negative effects combined with one positive effect, or (3) a combination of one positive, one negative, and one null effect.

### Which adolescents experience unity or duality?

To understand which adolescents experience within-person unity or duality in social media’s impact on the three mental health dimensions, we must consider risk and protective factors (World Health Organization, 2023). The differential susceptibility to media effects model (DSMM; Valkenburg & Peter, 2013) provides a valuable framework in this regard, as it posits that media effects vary based on individual susceptibility factors, which can either amplify or mitigate social media’s influence on well-being, self-esteem, and friendship closeness. Previous studies highlight the importance of examining the roles of age and gender, with girls and younger adolescents being more susceptible to negative impacts of social media on their mental health compared to boys and older adolescents, respectively (Fumagalli et al., 2024). Consequently, the groups of adolescents experiencing within-person unity or duality with predominantly or exclusively negative effects are likely to include more girls and younger adolescents compared to the groups experiencing predominantly or exclusively null or positive effects.

Similarly, adolescents’ self-concept clarity (SCC) serves as a susceptibility factor that may influence whether they experience within-person unity or duality. SCC reflects how clearly and confidently an individual’s self-concept is defined, and how consistent and stable it is over time (Campbell et al., 1996). Adolescents with high SCC are generally less influenced by their environment (Xu et al., 2023), and, consequently, may be less vulnerable to social media’s negative impacts. In contrast, those with low SCC may struggle with reconciling social media content with their unstable self-views (Appel et al., 2018), which may negatively affect their mental health (Quinones & Kakabadse, 2015). Thus, adolescents with lower SCC may experience more negative effects and duality (due to their unstable self-concept), while those with higher SCC may experience more null and positive effects. Therefore, we investigate how adolescents experiencing one of the three types of within-person unity or duality differ in terms of age, gender, and self-concept clarity (RQ2).

## Method

This preregistered study is part of a larger project examining the impact of social media use on adolescents’ mental health. The study procedure was designed following recommendations for collecting intensive longitudinal data (van Roekel et al., 2019). The full project was approved by the Ethics Review Board of the Faculty of Social and Behavioral Sciences at the University of Amsterdam (2022-YME-15724). The project was structured into four stages: (1) an online intake interview, (2) a baseline questionnaire, (3) a sequence of 100 daily micro-questionnaires, and (4) an optional exit interview. The current study focuses on data collected from the baseline questionnaire and the 100-day daily diary study. A detailed overview of the project can be found on OSF.

### Participants

The recruitment of participants was carried out in partnership with research company CHOICE, which had access to several panels comprising adults and adolescents over 16 who regularly took part in research studies. Adolescents interested in participating were encouraged to invite their friends to join the study. Additionally, participants were recruited from previous research projects, social media platforms, and the researchers’ personal networks. In line with the ethical standards of the Netherlands, informed consent was obtained from all adolescent participants as well as from parents or caregivers for those under the age of 16. Before providing consent, participants (and parents where applicable) received a detailed information letter and video explanations outlining the study procedure.

Initially, 480 adolescents aged 14 to 18 from various regions in the Netherlands enrolled in the larger project, but one adolescent withdrew after the first day of the daily diary study. Therefore, the final sample included 479 adolescents. The adolescents were enrolled in different educational tracks: vocational education (29.9%), higher general secondary/higher professional education (29.2%), and academic preparatory education (40.9%). Most of the adolescents were born in the Netherlands (96.9%).

### Procedure

In December 2022, participants were invited to an online intake interview and received instructions for installing the daily diary software application m-Path on their own smartphones. During the interview, participants learned about the study’s procedures and practiced filling out a sample questionnaire to get familiarized with the upcoming daily questions and response options.

Participants also selected their top three social media platforms, defined as online platforms where users can exchange information, such as TikTok, Instagram, Snapchat, WhatsApp, YouTube, and Discord, excluding platforms like Netflix and Spotify. We used this information to tailor the questions for each participant in the subsequent 100-day daily diary study. This approach aligns with research suggesting that adolescents typically engage with a small set of widely popular platforms alongside additional platforms specific to their personal interests (van der Wal et al., 2024), making a tailored, multi-platform approach ecologically most valid (Bayer et al., 2020; Frey & Friemel, 2023).

Five days before the 100-day diary study started, participants received a Qualtrics link to the baseline questionnaire, which included questions about demographics and self-concept clarity (SCC). From January to May 2023, participants received one questionnaire daily via the m-Path app for 100 consecutive days. The questionnaires were sent at 8:30 PM and could be started until midnight. If participants had not yet completed the questionnaire, reminders were sent at 9:15 PM and 10:00 PM. Each questionnaire consisted of 34–38 questions, depending on follow-up questions. At the end of the 100-day diary study, participants could extend their participation for 15 days to catch up on any missed days.

### Compliance and incentives

Participants’ compliance was monitored daily, and questions or issues were addressed via WhatsApp, telephone, and e-mail. To increase compliance, we also regularly contacted participants, for instance, to check for problems, update them on their weekly response rates, and reach out if they had missed three consecutive questionnaires. Over the 100-day period, 82.8% of the daily questionnaires were completed (39,598 out of 47,847 observations). After the additional 15 catch-up days, adolescents completed a total of 44,211 questionnaires, averaging 92.3 daily questionnaires per participant (SD = 24.55, range 12–115). A small fraction of the daily diaries (< 0.3%) encountered irregularities or were not sent due to unforeseen technical issues with the m-Path application. Non-responses were due to technological factors (e.g., uploading errors) and human factors (e.g., illness).

Participants received compensation for various parts of the study, excluding the optional exit interview. They were awarded €5 for the intake interview and another €5 for completing the baseline questionnaire. For each daily diary entry completed, participants earned €1. Those who completed all 100 questionnaires received an additional €10. Participants who completed 14 consecutive questionnaires during the middle phase of the study, from day 47 to day 60, earned a €5 bonus. Additionally, twice a week, two participants were randomly selected to receive a €25 compliance-based raffle prize. Compensation was distributed monthly throughout the study.

### Measures

#### Predictor (measured in diary study)

##### **Social media use**

Social media use was measured daily over the 100-day diary study. We personalized the diary questions to inquire about the time spent (in hours and minutes) on each participant’s three most frequently used social media platforms. Participants were instructed to consult their phone’s screen time data for accuracy. This method was chosen as a practical alternative to objective tracking, which was not feasible due to most participants using iPhones and the inability to install tracking software. A composite variable for social media use was constructed by summing the hours and minutes spent on each participant’s most used social media platforms. The total time spent, calculated in minutes, was used as the predictor variable in our analyses.

#### Outcomes (measured in diary study)

##### **Well-being**

We assessed daily affective well-being using a single adapted item from the Positive and Negative Affect Schedule for Children (PANAS-C; Ebesutani et al., 2012; Watson et al., 1988). Each evening, as part of the micro-questionnaire, adolescents answered the question “How happy did you feel today?” on a Visual Analog Scale (VAS) from 0 (not happy at all) to 100 (very happy). Previous research has shown consistent concurrent and convergent validity of this single-item happiness measure among adolescents (Lukoševičiūtė et al., 2022). Additionally, this approach has been effectively used in previous ESM studies assessing momentary affective well-being (Beyens et al., 2024; Karsay et al., 2023).

##### Self-esteem

We measured daily fluctuations in self-esteem using a single adapted item (Robins et al., 2001) from the Rosenberg Self-Esteem Scale (Rosenberg, 1986). Each evening, adolescents responded to the question “How satisfied with yourself did you feel today?” using a VAS from 0 (not satisfied at all) to 100 (very satisfied). Research has shown good psychometric properties of this single-item self-report measure of self-esteem (Atroszko et al., 2017; Robins et al., 2001). This measure has also proven effective in previous ESM studies in which momentary self-esteem was assessed (Valkenburg et al., 2021; Verbeij et al., 2022).

##### Friendship closeness

Every evening, adolescents were asked to rate their perceived closeness to their friends by answering the question “How close with your friends did you feel today?”, using a VAS ranging from 0 (*not close at all*) to 100 (*very close*). This approach is based on single-item measures as used in previous research (Bayer et al., 2016; Lee, 2009), which showed good psychometric properties (Atroszko et al., 2015), and previous ESM studies in which momentary friendship closeness was assessed (Pouwels et al., 2021). This single-item measurement is also consistent with previous daily dairy assessments of relationship quality (Battaglini et al., 2021; Lavee & Ben-Ari, 2007).

#### Individual difference variables (measured in baseline survey)

##### Gender

Gender was assessed using the statement “I am …” with three response options: (1) a boy, (2) a girl, and (3) fill in. Participants who selected “fill in” could provide their gender identity in a text box. The sample consisted of 44.3% boys, 54.9% girls, and 0.8% non-binary individuals.

##### Age

Age was calculated by subtracting the participant’s date of birth from the date the baseline survey was completed. The mean age was 15.98 years (SD = 1.15).

##### Self-concept clarity (SCC)

SCC was measured using the five-item Dutch version of the SCC scale (Valkenburg & Peter, 2008) adapted from the original twelve-item scale developed by Campbell et al. (1996) An example item is: “My beliefs about myself seem to change very frequently.” Participants responded on a 5-point Likert scale ranging from “not at all true” to “completely true.” The five items were inversely coded before calculating a mean score, with higher scores indicating a higher level of SCC. The mean score was 1.86 (SD = 0.83), and the scale demonstrated good internal consistency with a Cronbach’s alpha of 0.83.

To assess whether the SCC scale is unidimensional and could be used as a single variable in our analyses, we conducted both an exploratory factor analysis (EFA) and a confirmatory factor analysis (CFA). The EFA results supported a one-factor solution, χ² (5) = 8.98, *p* =.11, with all items loading significantly on a single factor and the single factor explaining 50.3% of the variance. CFA further confirmed the one-factor structure (Hu & Bentler, 1999): χ² (5) = 9.061, *p* =.107, CFI = 0.995, TLI = 0.991, RMSEA = 0.041 (90% CI: 0.000, 0.083), SRMR = 0.021, supporting that SCC is a unidimensional construct.

## Statistical analyses

The analyses were conducted in accordance with our preregistration. The syntax and data necessary to reproduce the analyses is also accessible on OSF.

We used dynamic structural equation modeling (DSEM) in Mplus 8.8. to estimate the effects of social media use on well-being, self-esteem, and friendship closeness. DSEM is an advanced modeling approach that integrates the strengths of multilevel analysis and Structural Equation Modeling (SEM) with *N* = 1 time-series analyses (McNeish & Hamaker, 2020). DSEM allows for the examination of the effects of social media use on well-being, self-esteem, and friendship closeness for individual participants, incorporating lagged (i.e., autoregressive) effects (Asparouhov et al., 2018; McNeish & Hamaker, 2020). This means that an individual’s well-being, self-esteem, and friendship closeness are regressed not only on their social media use but also on their prior day’s levels of well-being, self-esteem, and friendship closeness. This approach enables the interpretation of effects as within-person changes due to social media use. Additionally, DSEM analyses inherently control for all time-invariant third variables (e.g., socio-demographics).

Before estimating the DSEM models, we tested the required assumption of stationarity. Stationarity guarantees that the mean of well-being, self-esteem, and friendship closeness does not systematically change over the course of the study (McNeish & Hamaker, 2020). We inspected the explained variance of well-being, self-esteem, and friendship closeness (at the within-person level) by estimating a two-level fixed effect model including the day of study as a predictor of well-being, self-esteem, and friendship closeness. The assumption of stationarity was confirmed, as the day of the study explained only 0.3% of the within-person variance in well-being, 0.2% in self-esteem, and 0.0% in friendship closeness.

We used the DSEM default option for model estimation: Bayesian Markov Chain Monte Carlo (MCMC). Each DSEM model was run with a minimum of 5,000 iterations of the MCMC algorithm, with one-day time intervals. We estimated three two-level autoregressive lag-1 models (AR (1) models). At the within-person level (level 1), social media use was included as the predictor in each model (i.e., time-varying covariate), and well-being (Model 1), self-esteem (Model 2), and friendship closeness (Model 3) were included as the outcome variables. In each model, we used the social media use variable measured at the same time point as the outcome variable (i.e., from the same evening). Additionally, we included the autoregressive effect of each outcome, where each outcome was predicted by its previous measurement (lag-1). Latent person-mean centering was used to center the variables. At the between-person level (level 2), we included the latent mean level of each outcome variable and the latent mean of social media use.

Before interpreting the estimates, we checked model convergence (Hamaker et al., 2018). All three DSEM models converged after 5,000 iterations: The Potential Scale Reduction (PSR) was very close to 1 for all three models (PSR = 1.001), the density plots appeared smooth, and the trace plots did not show trends, spikes, or other irregularities (i.e., they resembled fat caterpillars) (Gelman & Rubin, 1992). Next, we doubled the number of iterations (i.e., a minimum of 10,000 iterations) to ensure the PSR value observed with 5,000 iterations was not close to 1 by chance (Schultzberg & Muthén, 2018). Since the results of the 10,000 iterations-models did not deviate from those of the 5,000 iterations-models, we proceeded with interpreting the estimates from the 5,000 iterations-models.

We interpreted the standardized effect sizes to determine whether the effects of social media use on well-being, self-esteem, and friendship closeness were positive, negative, and/or non-existent. For within-person associations, we considered an effect size of β = 0.05 as the smallest effect size of interest (SESOI) (Lakens et al., 2018), as this effect size is recommended for autoregressive studies (Adachi & Willoughby, 2015). Additionally, a recent meta-analysis (Meier & Reinecke, 2021) on the effects of social media on mental health confirmed that an effect size of 0.05 is relevant in the literature. Therefore, we interpreted within-person associations ranging from − 0.05 to + 0.05 as “non-existent to very small” and all associations beyond this range as negative or positive.

## Results

### Descriptive statistics and correlations

**Fig. 1:**
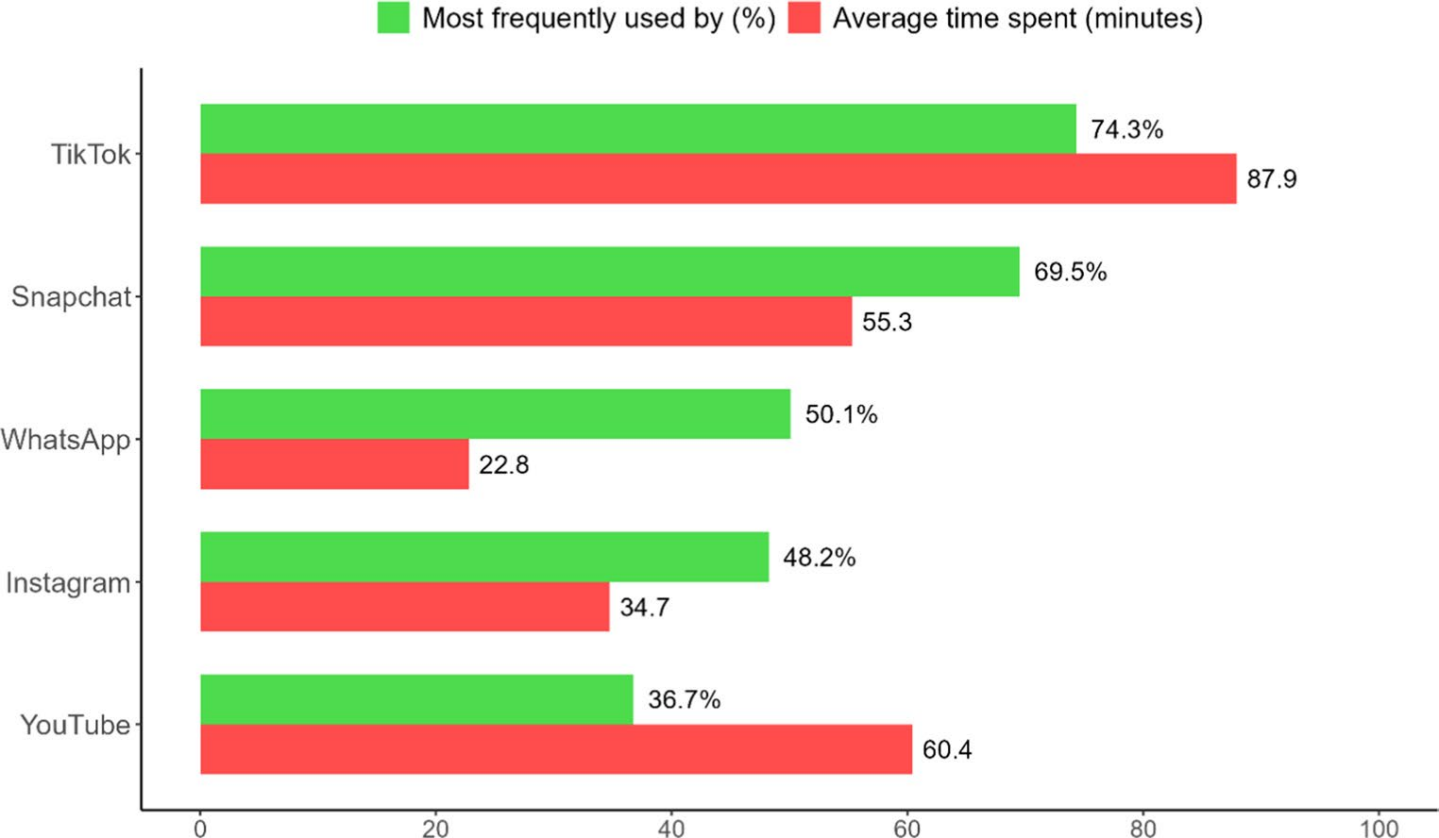
Adolescents’ most frequently used social media and average daily use per platform

Across all participants, 14 different social media platforms were listed, with a small subset (1.25%) reporting regular use of only two platforms. Figure 1 displays the five most frequently used platforms (the remaining nine were mentioned by fewer than 10% of adolescents), along with the average daily time spent on each. On average, participants spent 2 h and 40 min per day on their most frequently used platforms combined.

Table 1 presents the means, standard deviations, within-person, between-person, and intraclass correlations (ICCs) for social media use, well-being, self-esteem, and friendship closeness. The within-person correlations indicate that on days when adolescents reported higher well-being, they also tended to report higher self-esteem and closer friendships. The between-person correlations show that adolescents who reported higher overall well-being compared to their peers also reported higher self-esteem and stronger friendship closeness. The ICCs indicate that 41% of the variance in social media use, 39% in well-being, 37% in self-esteem, and 30% in friendship closeness is explained by differences between adolescents (i.e., between-person variance). These ICCs confirm the sampling scheme was appropriate for capturing within-person fluctuations in social media use and mental health and that the measures have sufficient within-person variance for DSEM analyses.

**Table 1:** Descriptive statistics and within-person, between-person, and intraclass correlations

	1	2	3	4
1. Social media use	—	− 0.06***	− 0.07***	− 0.05***
2. Well-being	− 0.08	—	0.43***	0.25***
3. Self-esteem	− 0.12*	0.82***	—	0.23***
4. Friendship closeness	− 0.01	0.61***	0.56***	—
M	160.55	72.37	65.46	64.30
SD	125.70	19.21	23.17	26.35
Range	0–1440	0–100	0–100	0–100
ICC	0.41	0.39	0.37	0.30

Mean scores for social media use reflect the average daily number of minutes spent using social media. Mean scores for well-being, self-esteem, and friendship closeness reflect adolescents’ average levels on a 0–100 scale. Correlations below the diagonal line represent between-person correlations and correlations above the diagonal line represent within-person correlations. ICC = intraclass correlation

*< 0.05. **< 0.01. ***< 0.001

### Person-specific effects of social media use

DSEM analyses revealed significant negative average within-person effects of social media use on the three dimensions of mental health, indicating that on days when adolescents spent more time on social media compared to other days, they experienced lower well-being (β = − 0.08, *p* <.001), self-esteem (β = − 0.09, *p* <.001), and friendship closeness (β = − 0.06, *p* <.001). When examining the distribution of person-specific effect sizes per mental health dimension, we found that 64.3% of adolescents experienced a negative effect on well-being, 24.4% experienced no effect, and 11.3% experienced a positive effect. For self-esteem, 71.8% of adolescents experienced a negative effect, 19.8% experienced no effect, and 8.4% experienced a positive effect. For friendship closeness, 58.0% of adolescents experienced a negative effect, 29.2% experienced no effect, and 12.7% experienced a positive effect.

### Within-person unity and duality

To assess the prevalence of within-person unity and duality in the effects of social media on well-being, self-esteem, and friendship closeness among adolescents (RQ1), we analyzed the person-specific effects across these three dimensions for each participant. Table 2 shows the distribution of adolescents across the three types of unity and duality. Around two-thirds of adolescents (68.1%) experienced unity in the impact of social media use on the three dimensions of mental health. This group predominantly consisted of adolescents experiencing unity in negative effects of social media use (59.5%). Approximately one in seven adolescents (13.6%) experienced a type of within-person duality, with the largest group exhibiting duality characterized by two negative effects and one positive effect (7.9%).

**Table 2:** Within-person unity and duality in person-specific effects of social media on mental health

Type of Unity or Duality	%	Gender Ratio	M Age (SD)	M SCC (SD)
Unity (neg)	59.5%	0.39	16.03 (1.12)	1.84 (0.84)
Unity (null)	3.6%	0.59	15.68 (1.29)	2.14 (0.69)
Unity (pos)	5.0%	0.58	16.02 (1.04)	1.98 (0.92)
Duality (2x neg, 1x pos)	7.9%	0.57	15.66 (1.24)	1.91 (0.66)
Duality (2x pos, 1x neg)	1.5%	0.71	16.40 (0.98)	2.06 (0.85)
Duality (1x pos, 1x neg, 1x null)	4.2%	0.45	16.02 (1.11)	1.87 (0.76)
Other (1x neg, 2x null)	12.7%	0.49	16.01 (1.20)	1.83 (0.85)
Other (1x pos, 2x null)	5.6%	0.54	15.85 (1.21)	1.81 (0.95)

“Neg” means negative effect, “null” means no effect, “pos” means positive effect. Negative and positive unity involve exclusively or predominantly uniform effects across dimensions and consequently may include one null effect. 88 adolescents (18.3%) experienced null effects on two dimensions and thus did not fall under a type of unity or duality (displayed as “Other”). Descriptives for age and SCC are based on a total *N* = 479. Because only four adolescents identified as non-binary, we could not include them as a separate group in our analyses. Therefore, the descriptives for gender are based on *n* = 475. The gender ratio represents the proportion of boys in each group

### Differences in gender, age, and SCC

To investigate whether adolescents displaying unity or duality in effects differed in terms of age, gender, and SCC (RQ2), we compared the six unity and duality groups. We found a statistically significant association between gender and the six unity and duality groups (*p* =.04) using Fisher’s Exact Test, which is more accurate for small sample sizes than the preregistered Chi-square test. However, pairwise comparisons between groups (adjusted for multiple comparisons using Bonferroni correction) did not show significant differences in the distribution of boys and girls across groups, likely due to large disparities in group sizes affecting the power of the tests. Additionally, two ANOVAs conducted to assess differences in age and SCC among the six groups revealed no significant differences. However, visual inspection of Table 2 suggests apparent trends, such as the negative unity group having slightly lower SCC scores and a higher proportion of girls compared to the positive and null unity groups. Yet, these potential trends should be interpreted with caution, as they could not be reliably tested in this study.

### Sensitivity analyses

As preregistered, we reran our DSEM models to ensure the robustness of our findings against potentially untrustworthy response patterns. These patterns included questionnaires with zero variance in responses or excessively high reported social media use, such as reporting over 20 h in a single day on a particular social media platform. Given that this study uses a composite score of time spent on multiple platforms, we also considered spending 20 h or more per day across platforms as untrustworthy. By excluding these untrustworthy responses, we removed 1,015 observations (2.3% of the total of 44,211 completed observations) from the analysis. Rerunning the DSEM models showed that the overall within-person effect sizes for the three dimensions of mental health, as well as the distribution of the effect sizes, remained unchanged.

### Person-specific effects across platforms

The number of adolescents experiencing negative effects of social media use on their well-being, self-esteem, and friendship closeness uncovered by the current study is much higher than reported in previous research (Beyens et al., 2024; Pouwels et al., 2021; Valkenburg et al., 2021). Therefore, we conducted exploratory analyses in which we ran the DSEM models for each of the top five platforms separately (i.e., TikTok, Snapchat, WhatsApp, Instagram, and YouTube) as some platforms may exert a particularly negative impact. As with the preregistered analyses for overall social media use, we examined the overall within-person effect sizes, the distribution of effect sizes, and the distribution of unity and duality. Table 3 provides the overall within-person effects of each platform on well-being, self-esteem, and friendship closeness. We found a consistent negative effect of time spent on TikTok, Instagram, and YouTube across all three mental health dimensions. Conversely, spending time on Snapchat positively affected friendship closeness and well-being, but had no significant effect on self-esteem. Using WhatsApp had a notably strong effect on friendship closeness, but no significant effect on well-being and self-esteem.

**Table 3:** Overall within-person effects of the top five social media platforms on mental health

	Well-being	Self-esteem	Friendship closeness
TikTok	− 0.09***	− 0.08***	− 0.09***
Instagram	− 0.05***	− 0.06***	− 0.04**
YouTube	− 0.08***	− 0.09***	− 0.11***
Snapchat	0.03***	0.01	0.09***
WhatsApp	− 0.00	0.01	0.17***

Statistically significant negative coefficients indicate overall negative within-person effects, statistically significant positive coefficients indicate overall positive within-person effects

*< 0.05. **< 0.01. ***< 0.001

**Fig. 2:**
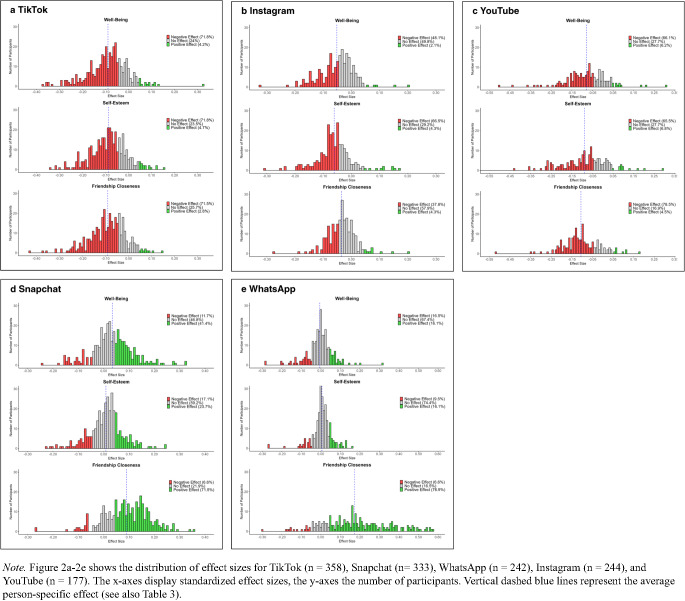
Distribution of person-specific effects of the top five social media platforms on mental health

Upon examining the distribution of person-specific effect sizes for well-being, self-esteem, and friendship closeness, we found striking differences across the top five social media platforms, as displayed in Fig. 2a-e. TikTok had a negative effect on all three dimensions among more than two-thirds of adolescents and a positive effect among less than 5% of adolescents. The results for YouTube were similar. For Instagram, we found slightly fewer negative effects and more null effects across dimensions compared to TikTok and YouTube, but a similarly low number of positive effects (less than 5%). In contrast, time spent on Snapchat positively impacted friendship closeness for 71.5% of adolescents, well-being for 41.4%, and self-esteem for 23.7%. Finally, WhatsApp impacted friendship closeness positively for more than three-quarters of adolescents, while a similarly large group experienced no effect on well-being and self-esteem.

### Within-person unity and duality across platforms

Table 4 reveals distinct patterns of within-person unity and duality across the different platforms. Compared to the distribution for overall social media use (59.5%), TikTok and YouTube users experienced more negative unity, with over two-thirds experiencing predominantly or exclusively negative effects on the three dimensions of mental health. Instagram users experienced less negative unity (51.1%) than TikTok and YouTube users, which seems to be due to more Instagram users (25.8%) experiencing one negative effect and two null effects. Positive unity was similarly low for these three platforms (less than 2.5%). In contrast, over one-fifth of WhatsApp users and nearly half of Snapchat users experienced positive unity. Additionally, nearly half of WhatsApp users showed one positive effect and two null effects.

**Table 4:** Within-person unity and duality in person-specific effects of the top five social media platforms on mental health

Type	TT (%)	SC (%)	WA (%)	IG (%)	YT (%)
Unity (neg)	71.0	7.5	5.0	51.1	66.7
Unity (pos)	1.4	45.7	21.1	1.3	2.3
Unity (null)	5.9	9.9	9.9	14.6	7.9
Duality (2x neg, 1x pos)	1.4	1.8	3.3	0.9	4.0
Duality (2x pos, 1x neg)	0.0	1.8	0.8	0.9	0.6
Duality (1x pos, 1x neg, 1x null)	3.9	5.7	8.7	2.2	5.1
Other (1x neg, 2x null)	13.1	6.6	5.4	25.8	12.4
Other (1x pos, 2x null)	3.4	21.0	45.9	3.4	1.1

*TT* TikTok (*n* = 358), *SC* Snapchat (*n* = 333), *WA* WhatsApp (*n* = 242), *IG* Instagram (*n* = 244), *YT* YouTube (*n* = 177). “Neg” means negative effect, “null” means no effect, “pos” means positive effect. Negative and positive unity involve exclusively or predominantly uniform effects across dimensions and consequently may include one null effect

## Discussion

The impact of social media on adolescent mental health remains a topic of intense debate. Some scholars argue that social media play a central role in the rise of mental health problems among youth (Haidt, 2020; Twenge et al., 2022), while others contend that the effects are minimal or even beneficial (Ferguson et al., 2025; Johannes et al., 2022). Rather than treating social media’s impact as universally positive or negative, the present study advances this debate by examining whether social media’s effects vary across multiple dimensions of adolescent mental health (well-being, self-esteem, and friendship closeness) within individual adolescents and across different platforms.

Leveraging an unprecedented dataset of 44,211 daily diary records from 479 adolescents collected across 100 days, we examined whether adolescents experienced uniform (unity) or diverging (duality) effects of social media on these three dimensions of their mental health. Worryingly, for 60% of adolescents, social media use had an exclusively or predominantly negative effect on well-being, self-esteem, and friendship closeness. For TikTok and YouTube use, this number was even higher (71% and 67%, respectively). These findings indicate that social media use is a contributor to mental health problems in the majority of adolescents.

### Within-person unity the dominant pattern

By using a novel framework centered around the concepts of within-person unity and duality, we found that 68% of adolescents experienced unity in either consistently negative, positive, or no effects across all three dimensions of mental health. Conversely, around one in seven adolescents exhibited within-person duality, indicating that social media use simultaneously harms and benefits different dimensions of their mental health. From a theoretical perspective, this approach offers a deeper understanding of how social media affect multiple dimensions of mental health within individual adolescents. Future research could build on this framework to investigate whether the impact of social media similarly aligns or diverges across additional mental health indicators, such as anxiety, stress, and emotion regulation, or even to physical health outcomes, including sleep quality and physical activity. Moreover, future work could extend the framework to other media environments such as gaming and streaming platforms, as these may likewise have uniform or diverging effects across different dimensions of mental health.

From a practical perspective, the presence of various forms of unity and duality underscores the need for personalized recommendations. To develop such recommendations, it is crucial to identify which adolescents are more likely to experience specific patterns of unity or duality in the effects of social media use. Therefore, guided by the differential susceptibility to media effects model (DSMM; Valkenburg & Peter, 2013), we examined whether gender, age, and self-concept clarity (SCC) explained the differential patterns. The skewed distribution – where 60% of adolescents fell into the negative unity group while the other groups remained relatively small – limited the robustness of statistical comparisons. Nonetheless, consistent with the DSMM, our findings suggest that girls were overrepresented in the negative unity group, indicating they may be particularly susceptible to consistently experiencing negative effects of social media use. Additionally, adolescents experiencing negative unity had slightly lower SCC compared to those experiencing null or positive unity, suggesting that self-concept clarity may serve as a protective factor against the negative effects of social media. Future research should further examine these patterns.

Future research should also investigate the role of other susceptibility factors in the impact of social media on mental health, guided by frameworks such as the DSMM (Valkenburg & Peter, 2013). One potential factor is an individual’s tendency to engage in upward social comparisons. For example, an experimental study found that adolescents with a strong tendency for social comparison were negatively affected by exposure to positively framed Instagram posts, whereas those without this tendency experienced positive effects (de Vries et al., 2018). Another possible factor is trait rumination, which involves responding to events with repetitive negative thinking and has been found to moderate the effects of daily events on negative affect (Chentsova et al., 2023; Genet & Siemer, 2012; Wang et al., 2018). Additionally, a recent umbrella review on social media use and adolescents’ mental health has highlighted several other individual difference variables that warrant further examination in future studies, such as socioeconomic status, the quality of parental relationships, and offline social networks (Sala et al., 2024).

### Social media’s impact differs between platforms

Our study is one of the first to systematically compare social media’s impact on adolescent mental health across different platforms. By analyzing the five most popular platforms in our sample – TikTok, Snapchat, WhatsApp, Instagram, and YouTube – we demonstrate that social media’s effects are not only person-specific but also platform-specific. TikTok, YouTube, and, to a lesser extent, Instagram, had a significant negative impact on all three dimensions of mental health. These highly visual platforms share several features that may help explain their adverse effects. First, they are saturated with edited, idealized images which are known to undermine adolescents’ self-esteem through upward social comparison processes (Rousseau & Rodgers, 2025). Second, these platforms’ algorithmic recommender systems often amplify exposure to distressing or problematic content, particularly for vulnerable youth (Hilbert et al., 2023). Third, their engagement-maximizing design often encourages excessive use, which can displace time and attention away from activities that support well-being, self-esteem, and friendship closeness.

In contrast, Snapchat and WhatsApp are primarily designed for private, reciprocal communication with known contacts. Their focus on ephemeral messaging and conversational exchange may foster social connection while limiting exposure to risks such as upward social comparison, algorithmic content overload, or displacement of social interactions. This may explain why Snapchat positively impacted well-being and friendship closeness, while WhatsApp strengthened friendship closeness without affecting well-being or self-esteem. These findings are further supported by Experience Sampling Method (ESM) studies that examined specific social media behaviors rather than platforms, which showed that social media activities such as messaging or posting are associated with higher social connectedness and well-being, whereas browsing and liking are linked to a decrease in mental health (Armstrong-Carter et al., 2023; Ferguson et al., 2024).

These platform differences highlight the need to move beyond a blanket condemnation of social media and instead focus on platform-specific risks and benefits. Interventions that aim to mitigate social media harms must be grounded in psychological theory and tailored to the specific ways in which platforms shape users’ experiences (Skeggs & Orben, 2025). Rather than focusing solely on screen time reduction, effective strategies should consider how different design features support or frustrate core psychological needs (Skeggs & Orben, 2025). For policymakers, this means regulating not just content but also design, for instance, by discouraging agency-reducing features like autoplay and infinite scroll, while promoting more need-supportive alternatives. A promising step in this direction are the European Commission’s draft guidelines on protecting minors under the Digital Services Act. For educators and parents, our findings underscore the importance of platform-specific digital literacy that helps adolescents understand how different platforms affect their mental health and fosters healthier usage habits.

### Understanding why social media’s negative impact appears more pronounced

Our study found a higher prevalence of negative effects compared to previous research using similar ESM approaches (Beyens et al., 2024; Karsay et al., 2023; Pouwels et al., 2021; Valkenburg et al., 2021). At least three explanations for this discrepancy are conceivable. First, the difference in the moment of assessment. In our 100-day study, participants completed a survey each evening, reflecting on their social media use and mental health for the entire day. In contrast, earlier ESM studies typically conducted assessments multiple times per day, focusing on the past hour (Beyens et al., 2024; Karsay et al., 2023; Valkenburg et al., 2021). Recent research has shown that negative impacts on mental health may not be immediate but instead emerge with a delayed onset (Klingelhoefer et al., 2024). It could be that initially, social media use may feel enjoyable or neutral, masking potential adverse effects that only become apparent upon reflection at the end of the day. Limited existing daily diary studies support this notion, finding negative effects of social media use on well-being (Colasante et al., 2024) and self-esteem (Irmer & Schmiedek, 2023).

At the same time, end-of-day assessments may be more susceptible to bias, as adolescents may recall the most intense or recent experiences (peak-end effect; Fredrickson, 2000). Moreover, negative experiences tend to carry more weight than positive or neutral ones (negativity bias; Rozin & Royzman, 2001). As a result, by the time adolescents reflect on their day in the evening, negative experiences may dominate their perceptions, leading to a heightened report of negative experiences relative to their actual momentary experience. Future studies should more closely examine how the timing and structure of assessments influence reported media effects, and consider methodological strategies to reduce recall distortions – such as combining self-reports with objective real-time behavioral data (Thomas, 2022). For instance, a sentiment analysis of social media activities or the valence of online social interactions could offer valuable insights into adolescents’ well-being, self-esteem, and friendship closeness during the day.

A third explanation for the increased negative effects observed in our study, compared to previous studies, could be the platforms we analyzed. For instance, ESM studies that found no effect on well-being (Beyens et al., 2024) and self-esteem (Valkenburg et al., 2021) used a composite score of time spent on Instagram, Snapchat, and WhatsApp. In our study, we found an overall negative effect for Instagram, but positive and null effects for Snapchat and WhatsApp. This could explain why those earlier studies found an overall null effect when combining the three platforms. In contrast, the daily diary study that found a negative effect on self-esteem used a composite score of time spent on Instagram, TikTok, and YouTube (Irmer & Schmiedek, 2023). This aligns with our findings, as we observed negative effects for each of these platforms individually, and we would expect a composite score to yield similarly negative results.

### Limitations and directions for future research

Of course, this study is not without limitations. While our 100-day intensive longitudinal design provides unique insights into the person-, dimension-, and platform-specific effects of social media use, several factors should be considered when interpreting our findings. First, we focused on adolescents aged 14 to 18, a critical developmental stage in which social media plays a central role. However, social media’s impact may differ for younger adolescents, emerging adults, or older age groups. Future research should explore other age ranges to examine how social media’s effects evolve across different developmental stages. Second, our study was conducted in a single WEIRD (Western, Educated, Industrialized, Rich, and Democratic) country, which may limit the generalizability of our findings. Future cross-cultural research is needed to determine whether these patterns hold across diverse sociocultural settings.

Third, to measure social media use, we relied on participants’ self-reported screen time. We instructed participants to check and report their phone’s recorded app usage data, likely improving accuracy over recall-based estimates. While we cannot confirm whether all participants followed this instruction consistently, research suggests that even recall-based self-reports have predictive validity comparable to digital trace data when assessing adolescent well-being, self-esteem, and friendship closeness (Verbeij et al., 2022), reinforcing confidence in our findings. Fourth, we measured social media use across each participant’s three most frequently used platforms, which enhances ecological validity but may not fully capture total engagement, potentially underestimating overall use and cumulative effects. As such, future research should consider alternative methodologies to deepen our understanding of how social media’s impact varies across individuals, platforms, and mental health dimensions.

Finally, although our DSEM models incorporated autoregressive effects and captured daily within-person fluctuations, they did not include time-varying contextual variables such as daily stressors or positive experiences. While we acknowledge the importance of considering contextual influences that may influence both adolescents’ social media use and their mental health on a given day, the current literature lacks consensus on which daily covariates are theoretically most relevant. Future research, for example using a network perspective (Aalbers et al., 2019), could help identify which contextual factors most strongly relate to social media use and mental health.

## Conclusion

The social media landscape has changed dramatically in recent years, most notably the rapid rise in TikTok’s popularity among youth. Additionally, well-established platforms have undergone major transformations, as seen with the introduction of YouTube Shorts and Instagram Reels, which have fundamentally altered how these platforms are used. The Instagram of today, for example, is vastly different from the platform studied just a few years ago, meaning previous findings may no longer fully represent its influence on young users. As such, our findings provide a crucial update on this evolving landscape and underscore the urgent need for ongoing research that takes into account these rapidly evolving platforms and their unique effects on youth mental health. By analyzing individual platforms separately, we emphasize that each has unique effects, and only through such specificity can we truly understand and mitigate the risks to adolescent mental health.

## Data Availability

The data underlying this article are publicly available on OSF: https://osf.io/p7ehy.

## Footnotes

1 This is a descriptive study, thereby limiting our ability to draw conclusions about cause and effect.

## Code availability

The preregistration of this study can be found on OSF: https://osf.io/ks9qa. The syntax necessary to reproduce the analyses is also accessible on OSF: https://osf.io/p7ehy. The code was checked and run by the third author, who was not involved in writing the code.
